# Inhibitory effects and mechanisms of proanthocyanidins against enterovirus 71 infection

**DOI:** 10.1016/j.virusres.2023.199098

**Published:** 2023-03-22

**Authors:** Jiqin Sun, Xiaoyao Ma, Lishan Sun, Yang Zhang, Cui Hao, Wei Wang

**Affiliations:** a Key Laboratory of Marine Drugs, Chinese Ministry of Education; School of Medicine and Pharmacy, Ocean University of China, 5 Yushan Road, Qingdao, 266003, P. R. China; b Laboratory for Marine Drugs and Bioproducts of Qingdao National Laboratory for Marine Science and Technology, Qingdao, 266237, P. R. China; c Medical Research Center, the Affiliated Hospital of Qingdao University, Qingdao, 266003, P. R. China

**Keywords:** Proanthocyanidins, Enterovirus 71, VP1 protein, Virus entry, MAPK pathway

## Abstract

Proanthocyanidins (PC), a natural flavonoid compound, was reported to possess a variety of pharmacological activities such as anti-tumor and anti-viral effects. In this study, the anti-Enterovirus 71 (EV71) activities and mechanisms of PC were investigated both *in vitro* and *in vivo*. The results showed that PC possessed anti-EV71 activities in different cell lines with low toxicity. PC can block both the adsorption and entry processes of EV71 via directly binding to virus VP1 protein. PC may competitively interfere with the binding of VP1 to its receptor SCARB2. PC can also regulate three different MAPK signaling pathways to reduce EV71 infection and attenuate virus induced inflammatory responses. Importantly, intramuscular therapy of EV71-infected mice with PC markedly improved their survival and attenuated the severe clinical symptoms. Therefore, the natural compound PC has potential to be developed into a novel anti-EV71 agent targeting viral VP1 protein and MAPK pathways.

## Highlights

• Proanthocyanidins (PC) possessed anti-EV71 activities *in vitro* with low toxicity.
• PC may be able to block the binding and entry processes of EV71 in RD cells.
• PC can bind to VP1 to interfere with the interaction between VP1 and SCARB2.
• The MAPK signaling pathways may be involved in the anti-EV71 actions of PC.
• PC can improve survival and attenuate clinical symptoms in EV71 infected mice.

## 1 Introduction

Enterovirus 71 (EV71), a non-enveloped RNA virus, belongs to the enterovirus genus of small RNA virus family (*Picornaviridae*). EV71 is one of the main pathogens causing hand, foot and mouth disease (HFMD) in infants, which affects the lives of millions of people, especially in the Asia Pacific region (Rasti et al., 2019). In some individuals, EV71 can infect the central nervous system, leading to serious life-threatening neurological diseases (Aswathyraj et al., 2016). However, until now, the pathogenic mechanism caused by EV71 is not clear, and there is no effective treatment for the diseases caused by EV71 (Wang et al., 2021). Thus, development of novel anti-EV71 drugs with high efficiency and low toxicity is urgently needed.

Proanthocyanidins (PC) belong to polyphenols, which widely exist in a variety of plants, and usually have many pharmacological activities, such as anti-oxidant, anti-inflammatory, anti-tumor, and anti-Alzheimer's disease effects (Chen et al., 2022; Gil-Cardoso et al., 2019; Rauf et al., 2019; Ruan et al., 2020; Swain et al., 2022; Zhao et al., 2019). Dai et al. reported that PC had inhibition on autophagy caused by influenza A virus (Dai et al., 2012). Maroli and coworkers have proved that PC may play a potential role in the treatment of COVID-19 by molecular dynamics simulation (Maroli et al., 2022). Fauvelle et al. found that PC may inhibit HCV entry most likely at a post-binding step (Fauvelle et al., 2017). Thus, PC has great potential to be developed as a novel anti-viral agent.

To further expand the anti-viral applications of PC, the anti-EV71 effects and mechanisms of PC were explored both *in vitro* and *in vivo* in this study. The results indicated that PC possessed anti-EV71 activities *in vitro* with low toxicity. PC can interfere with virus entry by acting on VP1 protein, and inhibit inflammatory responses via acting on MAPK pathways. Moreover, intramuscular administration of PC markedly attenuated the severe clinical symptoms and prevents death of EV71-infected mice. Thus, PC merits further studies as a novel entry inhibitor for EV71 in the future.

## 2 Materials and methods

### 2.1 Reagents, cells and viruses

Proanthocyanidins (purity ≥ 98%; PC) was obtained from Macklin Biochemical Co., Ltd. (Shanghai, China). Guanidine Hydrochloride was bought from sigma (Shanghai, China). Human embryonic rhabdomyosarcoma (RD) and Vero cells were grown in Dulbecco's modified Eagle's medium (DMEM) supplemented with 10% fetal bovine serum (FBS) (ExCell Bio, China), penicillin (100 U/mL), and streptomycin (100 μg/mL) at 37 °C in 5% CO2. EV71 strain BrCr-TR was obtained from the Wuhan Institute of Virology, Chinese Academy of Sciences.

### 2.2 Cytopathic effect (CPE) inhibition assay

The anti-EV71 activity was evaluated by the CPE inhibition assay (Smither et al., 2013; Dai et al., 2018). In brief, Vero or RD cells in 96-well plates were infected with PC (0.5, 1, 2, 4, 8 μM) pretreated (37 °C, 1 h) EV71 at a multiplicity of infection (MOI) of 0.1, respectively, and then treated with PC (0.5, 1, 2, 4 or 8 μM) in triplicate after virus adsorption. At 16 h post infection (p.i.), the cells were fixed with 4% formaldehyde for 15 min at room temperature (RT). Then the cells were stained with 0.1% (w/v) crystal violet for 30 min at RT. The plates were washed and dried, and the intensity of crystal violet staining for each well was measured at 570 nm.

### 2.3 Plaque reduction assay

Inhibition of EV71 infection by PC was also measured by the plaque reduction assay as described previously (Lin et al., 2019). EV71 (50–100 PFU/well) was pre-incubated with or without PC (0.0625–1 μM) for 60 min at 37 °C before infection. Then the virus-PC mixture was added to Vero cell monolayers in 6 or 12-well plates, and incubated at 37 °C for 60 min with gentle shaking every 15 min. After that, the inoculum was removed and each well was overlaid with 2 mL of agar overlay media containing 1.5% agarose. After incubation for 72 h at 37 °C, cells were fixed with 0.05% glutaraldehyde, followed by staining with 1% crystal violet in 20% ethanol for plaque counting.

### 2.4 Immunofluorescence assay

The indirect immunofluorescence assay of VP1 protein was performed as previously reported (Wang et al., 2017). In brief, for EV71 binding assay, EV71 (MOI = 5.0) was adsorbed to RD cells for 2 h at 4 °C with or without PC (20 µM) treatment before washing with PBS. For entry assay, EV71 (MOI = 5.0) was firstly adsorbed to RD cells at 4 °C for 2 h before washing with PBS. Then PC (20 µM) was added to cells and incubated at 37 °C for 2 h. Then cells were fixed, permeabilized, and incubated with primary antibodies against EV71 VP1 proteins (Immune Technology, Suzhou, China) and FITC-conjugated secondary antibodies (Boster, Wuhan, China), respectively. Finally, cells were washed and directly observed using Laser Scanning Confocal Microscope (Zeiss LSM 510, Jena, Germany). Images were analyzed by ImageJ (NIH) version 1.33 u (USA).

### 2.5 Western blot assay

After drug treatment, Vero cell lysates were separated by SDS-PAGE and transferred to nitrocellulose membrane. After being blocked in Tris buffered saline containing 0.1% (v/v) Tween 20 and 5% BSA overnight at 4 °C, the membranes were rinsed and incubated at 37 °C with antibodies against VP1 protein, cellular phosphorylated ERK, SAPK/JNK, p38MAPK, α-tubulin, GAPDH and β-actin proteins (Cell Signaling Technology, MA, USA), respectively. The membranes were then washed and incubated with AP-labeled secondary antibodies (Abbkine, USA) at RT for 2 h. Blots were developed with p-nitro blue tetrazolium chloride (NBT) and 5‑bromo-4‑chloro-3-indolyl phosphate toluidine (BCIP) solution at RT for 30 min. The relative densities of protein were analyzed by Image J (NIH) V.1.33 u (USA).

### 2.6 Pull down assay

For pull down assay, the cell lysates of pCDNA3.1-SCARB2-Myc transfected 293T cells in the radioimmunoprecipitation assay (RIPA) buffer (1% NP-40, 0.1% deoxycholate, 0.1% SDS, 150 mM NaCl, 10 mM Tris–HCl (pH 7.8), and 1 mM EDTA) containing a proteinase inhibitor cocktail (GE, USA), were incubated with anti-myc antibody conjugated magnetic beads (Sino Biological Inc., China) overnight at 4 °C before washing the beads three times with PBS containing 0.1% Tween 20 (v/v) (PBST). Then the EV71 particles was incubated with or without PC (100, 500 μM) at 37 °C for one hour. After that, the EV71-PC mixture was added to SCARB2-myc coupled magnetic beads and incubated at 4 °C for 2 h. After washed thrice with PBST, the VP1 protein bound to the beads was analyzed by immunoblotting with an anti-VP1 antibody, and the SCARB2-myc on the beads was detected by anti-myc antibody.

### 2.7 Surface plasmon resonance assay

SPR assays were conducted on a SPR biosensor instrument GE BiacoreT200 (GE, USA). EV71 VP1 protein (Abnova) was first immobilized onto the surface of a carboxymethylated dextran sensor chip (CM5) via amino group coupling. To assess real-time binding of PC to VP1 on CM5 chips, PC (50–3.125 μM) dissolved in DMSO was injected over the sensor chip surface within 2 min, followed by a 10 min wash with 1 × PBST buffer. The sensor chip surface was then regenerated by washing with NaOH (2 mM) for 30 s. All binding experiments were carried out at 25 °C with a constant flow rate of 2 μL/s PBS buffer. To correct for nonspecific binding and bulk refractive index change, a blank channel without VP1 was used and run simultaneously for each experiment. The changes in mass due to the binding response were recorded as resonance units (RUs).

### 2.8 Molecular docking analysis

Molecular docking was conducted in watvina software (http://www.biocheming.cn/). The crystal structure of EV71 VP1 protein (PDB code: 3VBH) was obtained from the protein data bank (http://www.rcsb.org), and processed in pyMOL software to export as PDBQT file for molecular docking. The 3D structure file of PC was obtained from PubChem database and imported into ChemBio3D Ultra 14.0 software for MM2 energy minimization before transformed into PDBQT file for molecular docking. The conformations with the lowest free energies of binding were selected as the best (probable) binding modes.

### 2.9 Mice experiments

The specific-pathogen-free ICR mice were purchased from Vital River Laboratory Animal Technology Co., Ltd. (Beijing, China), and maintained in a pathogen-free environment (23 ± 2 °C and 55% ± 5% humidity). All mice experiments were approved by the institutional animal care and use committee of the Ocean University of China (OUC-YY-202,201,001). One breeding ICR mouse could give birth to 12–13 pups, which were kept in a single group. Three-day-old neonatal ICR mice (*n* = 10–12 per group) were intraperitoneally challenged with EV71 at a dose of 10^5^ TCID_50_ per mice as reported (Zhang et al., 2017b) followed by an intramuscular injection of PC (5 or 10 mg/kg), Guanidine Hydrochloride (10 mg/kg) or PBS alone. Drugs were administered 12 h, 24 h and 48 h post-infection. The mice were monitored daily for 7 days to assess survival rate, clinical score and body weight. Signs of disease were evaluated by using a graded clinical score (0, health; 1, lethargy; 2, hind limb weakness; 3, single limb paralysis; 4, double limb paralysis; 5, dying or death).

### 2.10 Statistical analysis

All data are representative of at least three independent experiments. Data are presented as means ± standard deviations (SD). Statistical significance was calculated by GraphPad Prism 7.0 using one-way ANOVA analysis followed by post hoc Tukey's test. Statistical significance was considered to be *P* < 0.05.

## 3 Results

### 3.1 Inhibition of EV71 infection *in vitro* by PC

**Fig. 1:**
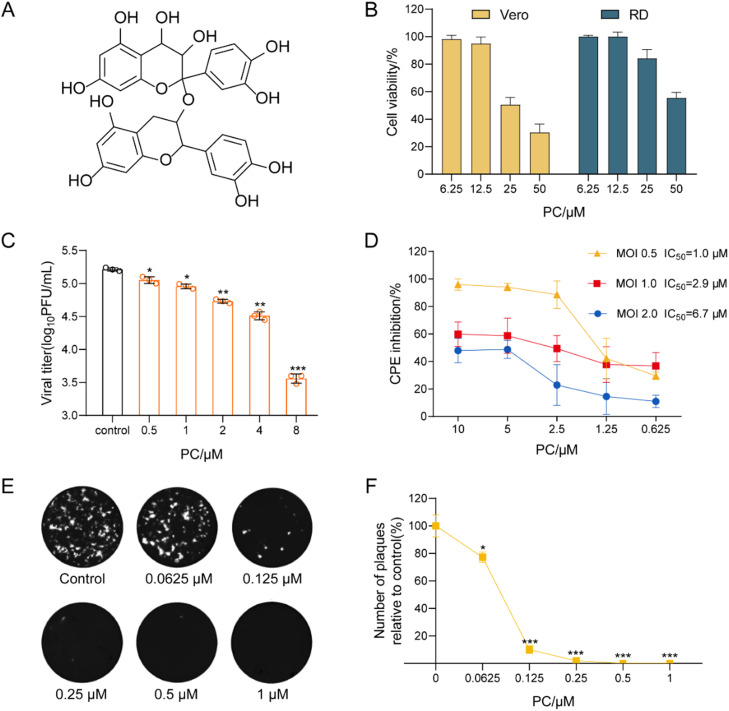
**Inhibitory effects of PC on EV71 infection *in vitro*.** (A) Schematic diagram of the chemical structure of PC. (B) The cytotoxicity of PC was determined by MTT assay. Values are means ± S*.*D. (*n* = 5). (C) The virus yields in EV71 (MOI = 0.1) infected cells at 16 h p.i. were determined by plaque assay. Values are means ± S*.*D. (*n* = 5). *P < 0.05, **P < 0.01, ***P < 0.001 vs. virus control group. (D) EV71 (MOI = 0.5, 1.0, or 2.0) infected Vero cells were treated with PC for 30 h, then the antiviral activity was determined by CPE inhibition assay. (E) Approximately 50–100 PFU/well of EV71 was pretreated with PC (0.0625–1 μM) for 60 min at 37 °C before infection. Then the virus-PC mixture was transferred to Vero cells, incubated at 37 °C for 1 h and subjected to plaque reduction assay. (F) Plaque numbers from plaque reduction assays performed on EV71 infected Vero cells (E). The percent inhibition was determined relative to the virus control. Values are means ± S*.*D. (*n* = 5). *P < 0.05, ***P < 0.001 vs. virus control.

Proanthocyanidins (PC) (Fig. 1A) was reported to possess a variety of pharmacological activities, so the inhibition of PC on EV71 infection was investigated in this study. Firstly, the cytotoxicity of PC in Vero and RD cells was evaluated by MTT assay (Li et al., 2020). PC exhibited no significant cytotoxicity at the concentrations from 6.25 to 50 μM in RD cells with the CC_50_ (50% Cytotoxicity Concentration) value of about 56.6 ± 2.7 μM. (Fig. 1B). In Vero cells, PC showed some cytotoxicity at the concentration of over 25 μM with the CC_50_ value of about 34.2 ± 1.4 μM (Table 1).

**Table 1:** Inhibitory effects of PC and Guanidine Hydrochloride on EV71 multiplication in different cells.

Compounds	Cell	CC_50_^1^	IC_50_^2^	SI (CC_50_/IC_50_) 3
PC (μM)	Vero	34.2 ± 1.4	0.8 ± 0.1	42.8
	RD	56.6 ± 2.7	1.5 ± 0.2	37.7
Guanidine Hydrochloride (μg/mL)	Vero	1689.9 ± 79.5	33.8 ± 6.5	49.9
	RD	1675.3 ± 12.5	102.7 ± 14.4	16.3

1 Cytotoxic concentration 50% (CC_50_): concentration required to reduce cell viability by 50%.

2 Inhibition concentration 50% (IC_50_): concentration required to reduce the CPE of the virus by 50% at 24 h p.i.

3 SI: Selectivity index is defined as the ratio of CC_50_ to IC_50_ (SI = CC_50_/IC_50_).

PC was then assayed for its anti-EV71 effects *in vitro* using plaque assay and CPE inhibition assay. Briefly, EV71 (MOI = 0.1) was pretreated with PC (0.5, 1, 2, 4, 8 μM) at 37 °Cfor 1 h before infection. Then after virus adsorption for 1 h, the virus inoculum was removed and the media with PC at the indicated concentrations were added into cells. After 24 h incubation, the cell viability was measured by CPE inhibition assay and the viral titers in the culture media were determined by plaque assay. The results indicated that PC significantly reduced the virus titers of EV7 in Vero cells when used at the concentration > 1 μM (*P* < 0.05) (Fig. 1C). CPE inhibition assay showed that the IC_50_ values of PC was about 0.8 ± 0.1 μM for EV71, and the selectivity index (CC_50_/IC_50_) for PC was about 42.8, comparable to that of Guanidine hydrochloride (SI = 49.9) (Table 1). PC also possessed inhibitory effect on EV71 in RD cells (SI = 37.7) (Table 1). Moreover, with the increase of MOI (MOI = 0.5, 1.0, 2.0), the IC_50_ values of PC increased correspondingly, but PC still possessed remarkable anti-EV71 activity when used at the concentration > 2.5 μM (Fig. 1D). In addition, pretreatment of EV71 with PC (0.0625–1 μM) at 37 °C for one hour significantly reduced the number of plaques in EV71 infected cells (Fig. 1E and F), suggesting that PC may be able to inactivate viral particles directly.

### 3.2 Influence of different treatment conditions of PC on EV71 infection

**Fig. 2:**
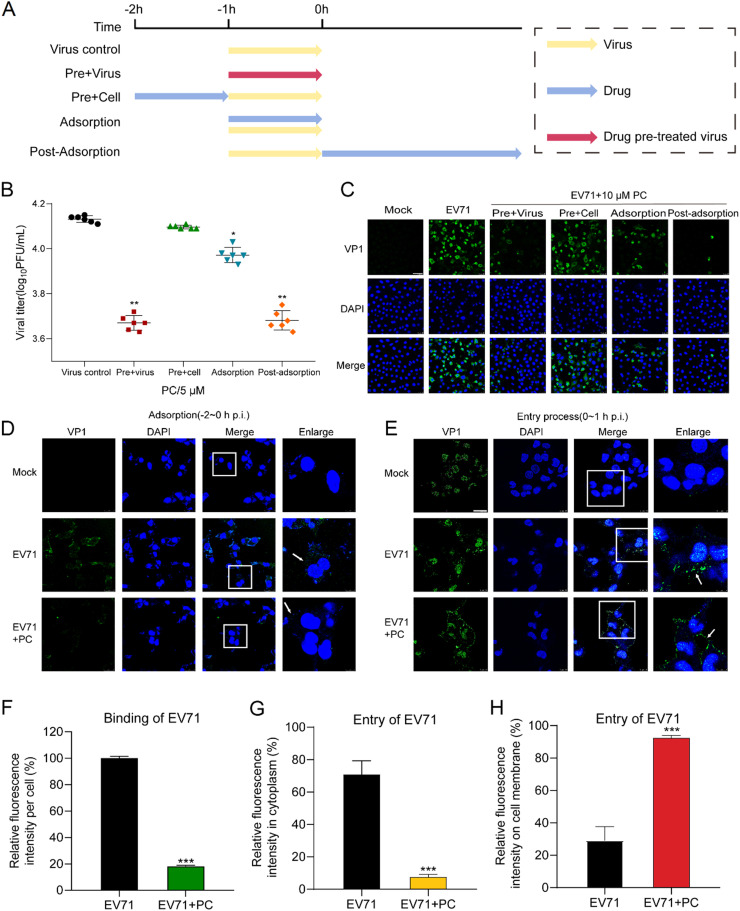
**Influence of different treatment conditions of PC on EV71 infection.** (A) Schematic diagram of the experiment processes in time of addition assay. (B) Time of addition assay: EV71 (MOI = 0.2) infected Vero cells were treated with PC (5 μM) under four different conditions: pre-treatment of viruses, pre-treatment of cells, during virus adsorption, or post adsorption. At 24 h p.i., virus yields were determined by plaque assay. Values are means ± S*.*D. (*n* = 3). *P < 0.05, **P < 0.01 vs. virus control group. (C) Vero cells were infected with EV71 (MOI = 0.2) under four treatment conditions of PC (10 μM), and the expression of VP1 was detected by immunofluorescence assay. Scale bar represents 50 μm. (D and E) EV71 (MOI = 5.0) infected RD cells were treated with or without PC (20 μM) during adsorption (D) or entry process (E), then after washing, the immunofluorescence assay of VP1 was performed. Scale bar represents 20 μm. (F-H) The average fluorescence intensity of VP1 during adsorption (F) or entry process (G) was measured by Image J (NIH) version 1.33 u (USA) to calculate the average intensity per cell of different images (*n* = 10). The average intensity of VP1 on cell membrane during entry (H) was also measured by Image J (USA). ***P < 0.001 vs. virus control group (EV71).

To explore the stage (s) at which PC exerted its inhibition actions *in vitro*, the time-of-addition assay was performed as described previously (Li et al., 2020). Briefly, PC was added to EV71 (MOI = 0.1) infected Vero cells under four different conditions: pre-treatment of viruses, pre-treatment of cells, during virus adsorption, or post adsorption (Fig. 2A). At 24 h p.i., the anti-EV71 activity was determined by plaque assay. As shown in Fig. 2B, pretreatment of EV71 with 5 μM PC significantly reduced the virus titers of EV71, as compared to the virus control group (*P* < 0.01), suggesting that PC may have direct interaction with EV71 particles. The addition of PC during adsorption or post adsorption also possessed significant inhibition on EV71 multiplication (*P* < 0.05) (Fig. 2B). Moreover, the immunofluorescence assay also indicated that pretreatment of EV71 with PC (10 μM) before infection or addition of PC after virus adsorption all significantly decreased the expression of VP1 in EV71 infected cells (Fig. 2C), consistent to the results of plaque assay (Fig. 2B). Thus, PC may be able to interact with EV71 particles to block virus adsorption or inhibit some steps after virus adsorption.

Since PC may inhibit adsorption and post-adsorption processes of EV71, we further explored whether PC can directly block the binding (−2∼0 h p.i.) and entry of EV71 (0∼1 h p.i.) by using immunofluorescence assay. The results indicated that PC treatment (20 μM) during adsorption (−2∼0 h p.i.) significantly reduced the fluorescence of VP1 on cell surface, suggesting that PC can block the adsorption process of EV71 (Fig. 2D and F). Interestingly, at 1 h p.i., lots of fluorescence spot of VP1 located at the nucleus area or cytoplasm of virus control cells, suggesting that most of EV71 particles had entered into cytoplasm (Fig. 2E). However, PC treatment (20 μM) dramatically reduced the fluorescence of VP1 in cytoplasm, and lots of fluorescence spot was found on cell surface (white arrow indicated) (Fig. 2E, G and H), suggesting that PC may also block EV71 entry process. Taken together, PC may block EV71 infection mainly via interfering with virus binding and entry processes.

### 3.3 PC may interfere with EV71 infection through targeting VP1 protein

**Fig. 3:**
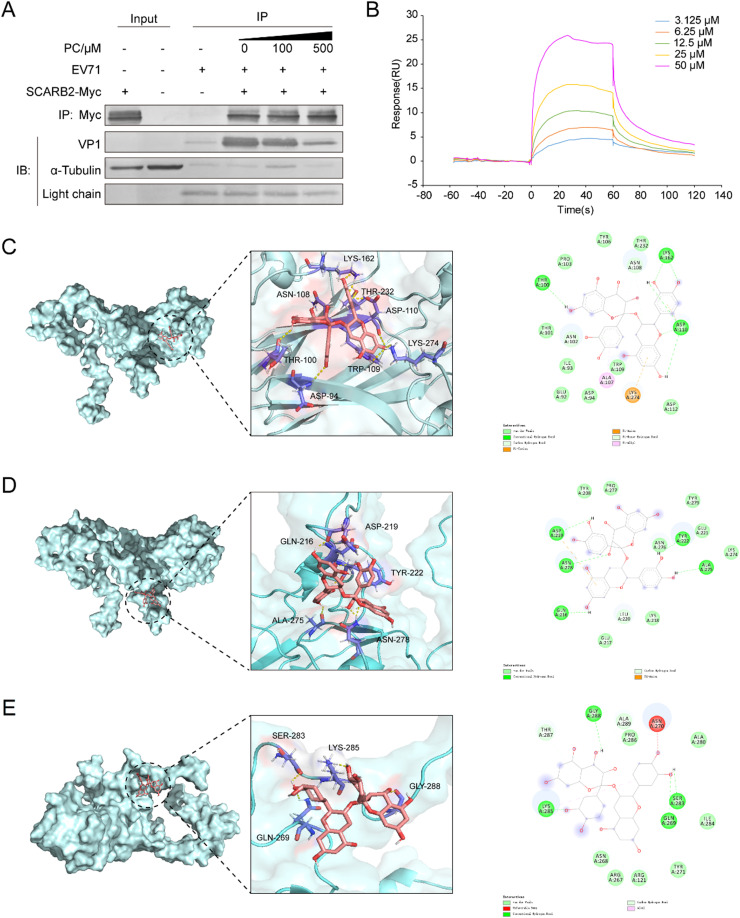
**PC may interfere with EV71 infection through targeting VP1 protein.** (A) The cell lysates of pCDNA3.1-SCARB2-Myc transfected RD cells were incubated with anti-myc magnetic beads overnight at 4 °C before washing the beads three times with PBST. Then the PC (100, 500 μM)-EV71 mixture was added to SCARB2-myc coupled magnetic beads and incubated at 4 °C for 2 h. After washed thrice with PBST, the VP1 and SCARB2-myc proteins on the beads was detected by western blot. (B) The EV71 VP1 proteins were immobilized onto the surface of a CM5 sensor chip. DMSO was first used to flow through the chip surface to reset in order to remove the influence of DMSO solvent. PC (50, 25, 12.5, 6.25, 3.125 μM) was then flowed over the biosensor chip surface. The sensorgram for all binding interactions were recorded in real time and the changes in mass due to the binding response were recorded as resonance units (RU). (C-E) The 2D and 3D binding modes of PC with the three binding pockets (Glu92-Tyr106, Gly212-Met225, Ala280-Ala293) of VP1 (PDB ID: 3VBH) for SCARB2 binding were shown, respectively. PC is colored in red, the surrounding residues are colored in purple. The backbone of the receptor is depicted as cyan cartoon.

Scavenger receptor B2 (SCARB2) is reported to be the main functional entry receptor for EV71 in most cells (Zhou et al., 2019), and VP1 protein can interact with SCARB2 to facilitate both the virus internalization and RNA uncoating of EV71 (Dang et al., 2014). Thus, we further explored whether PC can interfere with the binding of EV71 to its receptor SCARB2 by performing pull-down assay. Briefly, the cell lysates of pCDNA3.1-SCARB2-Myc transfected 293T cells were incubated with anti-myc antibody conjugated magnetic beads before incubation with EV71-PC (100, 500 μM) mixture. Then the VP1 and SCARB2-myc proteins bound to the beads were detected by western blotting. The results showed that the SCARB2-myc protein can specifically bind to the magnetic beads and lots of VP1 proteins bound to SCARB2-myc beads could be detected (Fig. 3A). However, PC (100, 500 μM) pretreatment of EV71 particles dose-dependently reduced the amount of VP1 bound to SCARB2-myc beads (Fig. 3A), suggesting that PC can truly interfere with the binding of EV71 particles to virus entry receptor SCARB2.

To further explore whether PC can directly bind to VP1 protein, the SPR assay was performed as reported previously (Li et al., 2020). Briefly, with EV71 VP1 proteins being immobilized on the chip, PC at the concentrations of 3.125–50 μM was flowed over the biosensor chip surface, respectively. Data revealed a marked binding of PC to virus VP1 protein in a concentration-dependent manner with a KD equivalent to about 10.1 μM, implicating a high affinity of PC for VP1 (Fig. 3B). Thus, pretreatment of EV71 with PC before infection may allow PC to fully bind VP1 and form a stable PC-VP1 complex to block virus adsorption.

It has been shown that the 92–106, 212–225 and 280–293 amino acid sequences of VP1 protein are three main active sites for its binding to SCARB2 (Ku et al., 2015). To further investigate whether PC can bind to these three active sites of VP1 protein, the molecular docking analysis was carried out using the crystal structure of EV71 VP1 protein (PDB code: 3VBH). The docking analysis between PC and the first active pockets (Glu92-Tyr106) indicated that PC can form eight hydrogen bonds with VP1 protein (Asp94, Thr100, Asn108, Trp109, Asp110, Lys162, Thr232 and Lys274). Similarly, PC can also form six hydrogen bonds with the second active sites (Gly212-Met225) of VP1 (Gln216,Asp219,Tyr222,Ala275 and Asn278), and form four hydrogen bonds with the third active sites (Ala280-Ala293) of VP1 (Gln269,Ser283,Lys285 and Gly288) (Fig. 3C-E). The interaction energy between PC and these three active sites in VP1 was about −9.19, −7.36 and −7.14 kcal/mol, respectively, suggesting that PC may specifically bind to the active pockets of VP1 which are required for the binding to SCARB2. Thus, PC may block EV71 entry mainly through binding to VP1 to interfere with the interaction between VP1 and SCARB2.

### 3.4 Transcriptome analysis identifies the differential expressed genes in EV71-infected cells

**Fig. 4:**
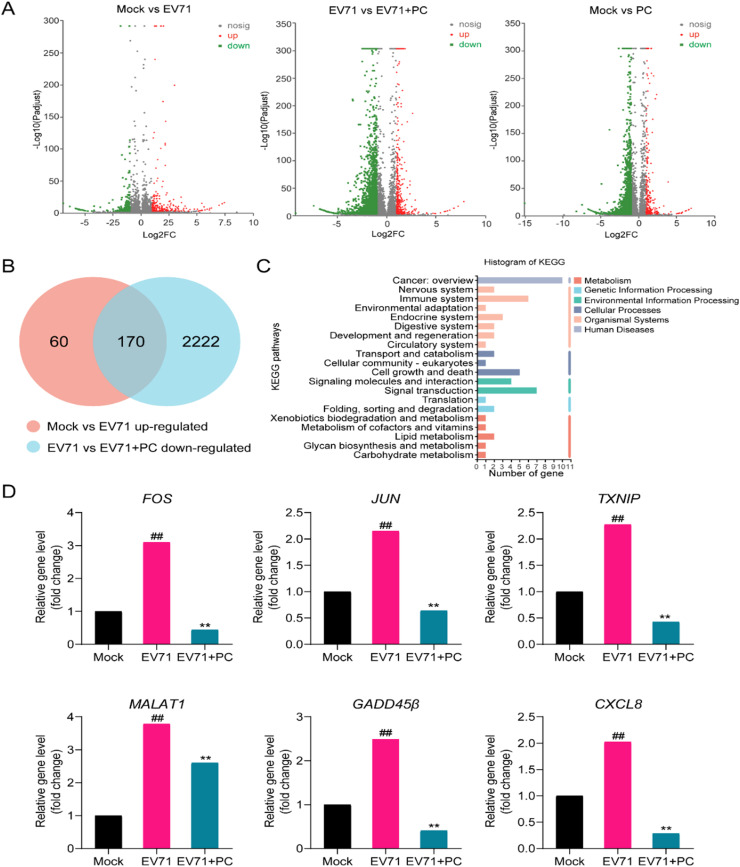
**Transcriptome analysis identifies the differential expressed genes in PC treated EV71-infected cells.** (A) The EV71 (MOI = 0.5) infected or non-infected RD cells were treated with or without PC (10 μM) for 10 h after virus adsorption. Then the purified RNAs isolated from four experimental groups (Mock, PC, EV71, EV71+PC) were subjected to transcriptome analysis. Volcano plots indicate up-regulated (red) and down-regulated (green) mRNA transcripts in the Mock *vs* EV71 group, EV71 *vs* EV71+PC group or Mock *vs* PC group. (B) Venn analysis indicates about 170 differentially expressed genes (DEGs) are up-regulated in the Mock *vs* EV71 group and also down-regulated in the EV71 *vs* EV71+PC group. (C) The KEGG annotation analysis was performed to display the 170 DEGs related biological processes in EV71 infected cells (Mock *vs* EV71). (D) Some DEGs with up-regulation after EV71 infection (log2FC(EV71/Mock)>1) in RD cells (*P* < 0.01) were shown. The relative gene levels of *FOS, JUN, CXCL8, GADD45β, TXNIP* and *MALAT1* were calculated by SAM analysis. The relative RNA levels for non-infected control cells (Mock) were assigned values of 1.0. Values are means ± SD (*n* = 3). ##*P* < 0.01 vs. mock control group; **P < 0.01 vs. virus control group (EV71).

The results of the time of addition assay indicates that PC may also inhibit some steps after virus adsorption, so we further performed the transcriptome analysis in order to elucidate the possible target of PC in EV71-infected RD cells (Yao et al., 2019). Briefly, the EV71 (MOI = 0.5) infected or non-infected RD cells were treated with or without PC (10 μM) for 10 h after virus adsorption. Then the purified RNAs isolated from four experimental groups (Mock, EV71, PC, EV71+PC) were subjected to cDNA libraries construction before sequencing on an Illumina HiSeq Xten platforms by Majorbio technology Inc. Differential gene expression was calculated by SAM analysis. The results showed that there were about 230 upregulated genes and 170 downregulated genes were identified (fold change ≥ 2 and *P* < 0.001) in virus control group (EV71) relative to the non-infected group (Mock) (Fig. 4A). However, after PC (10 μM) treatment, there were about 356 upregulated genes and 2392 downregulated genes were identified in PC treated infection group (EV71+PC) relative to the virus control group (EV71) (Fig. 4A). Among them, about 170 upregulated genes in the Mock *vs* EV71 group were down-regulated in the EV71 *vs* EV71+PC group (Fig. 4B), indicating that these 170 genes may be related to the inhibition effect of PC on EV71 infection in RD cells. Moreover, the KEGG annotation analysis was performed to further display the DEGs related biological processes. The results showed that the “Cancer:overview”, “Immune disease”, “Signal transduction”, “Immune system” and “Infectious disease” are the most highly represented in terms of both -log10 (FDR) and number of genes (> 140) (Fig. 4C).

To further explore the possible anti-EV71 targets of PC, we screened the DEGs with up-regulation after EV71 infection (log2FC(EV71/Mock) > 1) and down-regulation after PC treatment (log2FC(EV71/Mock) ≤ 0.5) in RD cells (*P* < 0.01), and six DEGs related to inflammation and apoptosis were obtained in our study (Fig. 4D). Among them, EV71 induced inflammation related genes *FOS, JUN, CXCL*8 (17–19), and virus induced apoptosis related genes *GADD45β, TXNIP,* and *MALAT1* (16, 20, 21) were all significantly up-regulated in virus control group (EV71), and down-regulated after PC treatment (EV71+PC) (*P* < 0.01) (Fig. 4D). Considered that these DEGs are mainly involved in virus induced inflammatory response and cell apoptosis, we speculate that PC may reduce the expression of these DEGs to attenuate EV71 induced inflammatory responses and cell apoptosis.

### 3.5 Inhibition effects of PC on EV71 induced inflammatory responses

**Fig. 5:**
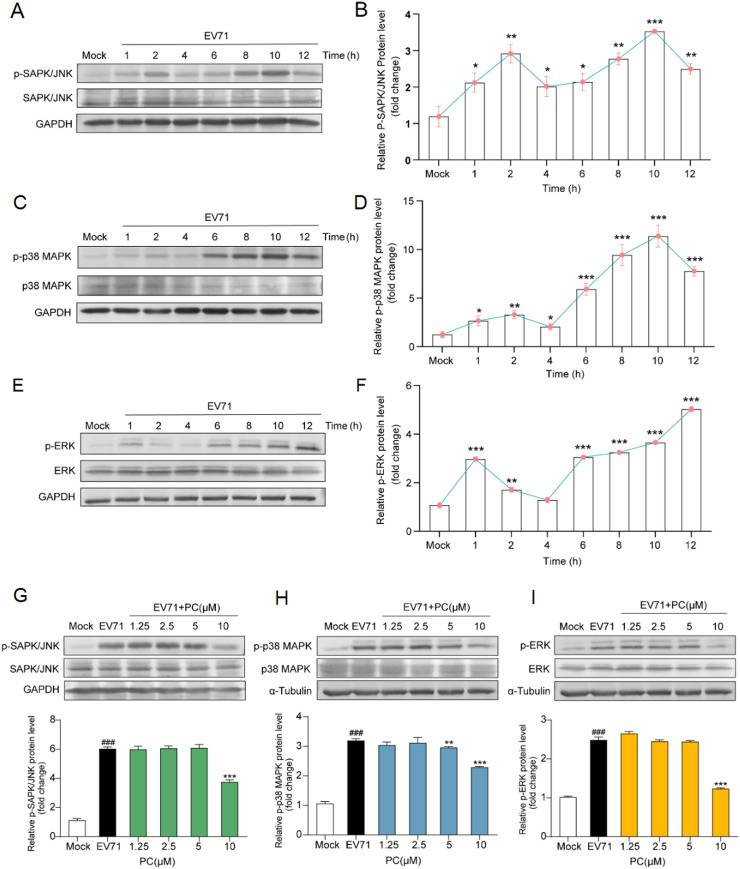
**Inhibition effects of PC on EV71 induced inflammatory responses.** (A-F) EV71 (MOI = 1.0) infected Vero cells, and the cell lysates were collected at different time points post infection (1 h, 2 h, 4 h, 6 h, 8 h, 10 h, 12 h p.i.). Then the phosphorylation and total levels of SAPK/JNK (A), p38MAPK (C) and ERK (E) was evaluated by western blot. Quantification of immunoblot for the ratio of p-SAPK/JNK (B), p-p38MAPK (D) or p-ERK (F) to GAPDH was also shown. (G-I) EV71 (MOI = 1.0) infected Vero cells were treated with or without PC (1.25, 2.5, 5, 10 μM) for 10 h. Then the levels of phosphorylated and total SAPK/JNK (G), p38MAPK (H) and ERK (I) were detected by western blotting. Blots were also probed for α-tubulin or GAPDH proteins as loading controls. Quantification of immunoblot for the ratio of SAPK/JNK (G), p38MAPK (H) and ERK (I) to α-tubulin or GAPDH, respectively. The ratio for non-infected cells (Mock) was assigned values of 1.0. Values are means ± S*.*D. (*n* = 3). ###*P* < 0.001 vs. normal control group (Mock); *P < 0.05, **P < 0.01, ***P < 0.001 vs. virus control group (EV71).

Since the transcriptome analysis suggests that some DEGs involved in inflammatory response may be the main targets of PC after virus adsorption, we further explored if PC could influence the signaling pathways required for EV71 infection and virus induced inflammatory responses by western blotting. It has been reported that EV71 infection can induce the production of ROS to lead to the phosphorylation of p38MAPK, ERK and JNK, thereby promoting EV71 replication in host cells (Jin et al., 2018; Li et al., 2015; Liao et al., 2019; Liu et al., 2019; Lu et al., 2021; Peng et al., 2014). Thus, we first explored the activation levels of MAPK signals at different time points after EV71 infection. As shown in Fig. 5A-F, the expression levels of phosphorylated SAPK/JNK, p38MAPK and ERK proteins were elevated in varying degrees with the infection of EV71 (MOI = 1.0). Among them, the expression levels of p-SAPK/JNK were slightly increased at 2 h p.i., and strongly reactivated at 8 h p.i. (Fig. 5A and B). Similarly, the phosphorylation of p38MAPK was obviously increased at 6 h p.i., and its activation state lasted over six hours followed by decrease at 12 h p.i. (Fig. 5C and D). Moreover, the levels of p-ERK could be slightly increased at 1 h p.i., and strongly reactivated at 6 h p.i. (Fig. 5E and F).

After that, we further evaluated the influence of PC on MAPK pathways in EV71 infected cells at 10 h p.i. As shown in Fig. 5G-I, PC (10 μM) treatment significantly reduced the levels of p-SAPK/JNK, p-p38MAPK and p-ERK at 10 h p.i., as compared to the virus control group (*P* < 0.001) (Fig. 5G-I). PC (5 μM) treatment also significantly reduced the phosphorylation of p38MAPK at 10 h p.i. (Fig. 5H). Therefore, PC may also have inhibition on three different MAPK signaling pathways to reduce EV71 infection and attenuate virus induced inflammatory responses.

### 3.6 PC also possessed *in vivo* antiviral activity in EV71 infected mice

**Fig. 6:**
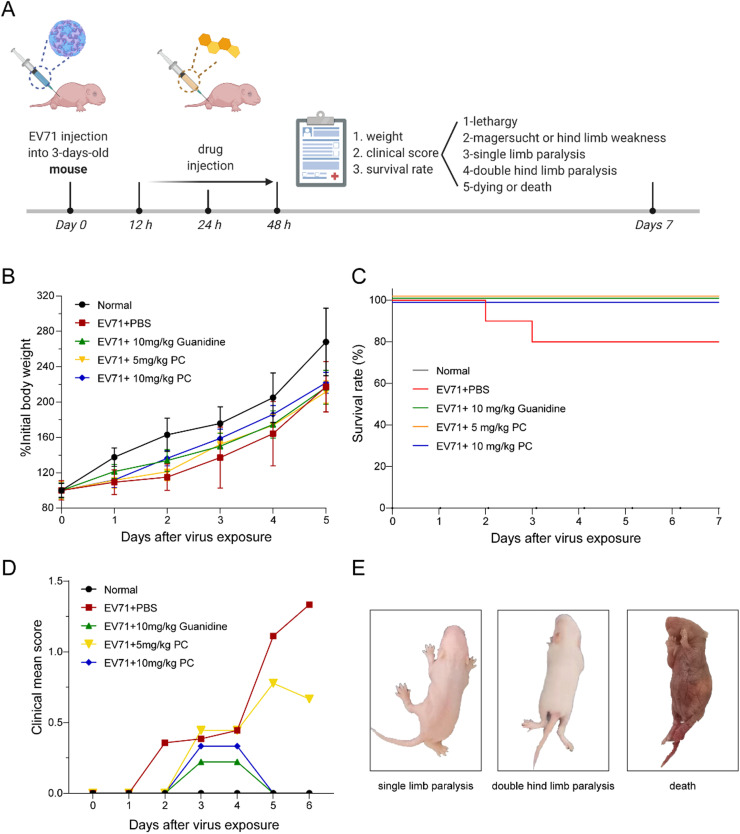
**PC also possessed anti-EV71 activities *in vivo*.** (A) The EV71 infected neonatal mice model was established by intraperitoneal injection of EV71 into three-day-old ICR mice and drug treatment was given by intramuscular injection at 12 h, 24 h and 48 h p.i. Created with BioRender.com. (B) Body weights. The average body weights in each group were monitored daily for 8 days and expressed as a percentage of the initial value. (C) Survival rate. EV71 infected mice were received intramuscular treatment of PC (5 or 10 mg/kg), Guanidine Hydrochloride (10 mg/kg), or PBS at 12, 24 and 48 h after EV71 infection. Results are expressed as percentage of survival, evaluated daily for 8 days. (D) The clinical scores indicating the clinical manifestations of infected mice in each group were monitored daily for 6 days. (E) Three different clinical manifestations: single limb weakness, double hind limb weakness and death.

The anti-EV71 activities of PC were further tested in a newborn mice model. Three-day-old ICR mice were intraperitoneally injected with EV71 at a dose of 10^5^ TCID_50_ per mice followed by intramuscular administration of drugs at 12, 24 and 48 h after exposure (Fig. 6A). The results showed that the body weights of infected mice treated with PC (5 or 10 mg/kg) increased faster than that of the virus control group (EV71+PBS) until the end of the observation period (Fig. 6B). PC treatment also markedly improved the survival of infected mice (100% survival) as compared to the virus control group (80% survival), similar to the effect of Guanidine Hydrochloride (10 mg/kg) (100% survival) (Fig. 6C).

Moreover, after EV71 infection, the ICR mice showed some clinical manifestations, including drowsiness, weight loss, single limb weakness, double hind limb weakness and death (Fig. 6E). Compared to the virus control group (EV71+PBS), PC treatment (5 or 10 mg/kg) dramatically reduced the clinical scores of EV71 infected mice (Fig. 6D), consistent to the protective effects of PC on survival. PC-treated (10 mg/kg) infected mice only showed some symptoms of lethargy, without severe symptoms such as limb paralysis or death within eight days. 5 mg/kg of PC or 10 mg/kg of Guanidine Hydrochloride treated infected mice only showed slight limb paralysis but all survived. Thus, PC can also protect newborn mice from lethal EV71 challenge.

## 4 Discussion

Proanthocyanidins (PC), the natural flavonoid compound, were reported to possess a variety of pharmacological activities (Rauf et al., 2019; Chen et al., 2022). In this study, we discovered that PC possessed anti-EV71 activities with low toxicity in different cell lines. PC may block EV71 infection via binding to VP1 protein to interfere with the interaction between VP1 and its receptor SCARB2. PC may also have inhibition on MAPK pathways to reduce EV71 infection and attenuate virus induced inflammatory responses. Importantly, intramuscular therapy of EV71-infected mice with PC markedly improved their survival and attenuated the severe clinical symptoms, suggesting that PC has potential to be developed into a novel anti-EV71 agent.

**Fig. 7:**
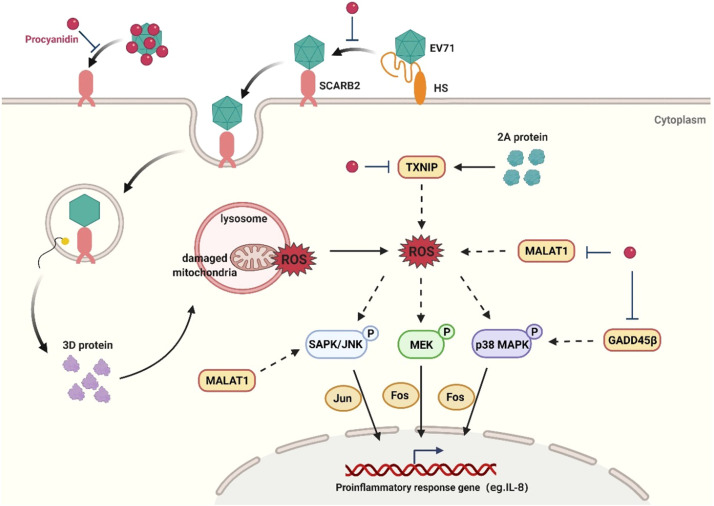
**A proposed model underlying the anti-EV71 mechanisms of PC.** PC can block EV71 adsorption and entry via targeting VP1 protein to interfere with the binding of VP1 to its receptor SCARB2. PC can also inhibit cellular MAPK pathways to attenuate virus infection and inflammatory responses. Created with BioRender.com.

The EV71 virion is composed of 60 capsid copies, each of which is made up of four capsid proteins: VP1, VP2, VP3 and VP4 (Plevka et al., 2012; Zhang et al., 2017a). Among them, VP1 protein has been shown to interact with the main cell surface receptor scavenger receptor B2 (SCARB2) (Zhou et al., 2019) to facilitate both the internalization and RNA uncoating of EV71 (Dang et al., 2014). Herein, we found that PC can directly interact with EV71 VP1 protein to interfere with the binding of EV71 to its receptor SCARB2 (Fig. 3). The molecular docking analysis indicated that PC may bind to three pockets (Glu92-Tyr106, Gly212-Met225 or Ala280-Ala293) of VP1 required for SCARB2 binding so as to block the entry of EV71. Furthermore, the transcriptome analysis suggested that some DEGs such as *FOS* and *JUN* related to inflammatory response may be the targets of PC. Western blot assay verified that PC can truly down-regulate cellular MAPK pathways which are required for viral replication and inflammatory responses (Jin et al., 2018; Li et al., 2015; Liao et al., 2019; Liu et al., 2019; Lu et al., 2021; Peng et al., 2014). Thus, PC may exert its anti-EV71 effects mainly through direct interaction with VP1 to block virus entry and indirect regulation of MAPK pathways to further reduce EV71 infection and inflammatory responses (Fig. 7).

The *in vitro* anti-EV71 effects of PC were mirrored in EV71-infected newborn mice. Herein, we found that intramuscular therapy of EV71-infected mice with PC markedly improved their survival and increased the body weights of EV71 infected mice (Fig. 6). PC treatment significantly attenuated the severe symptoms such as limb paralysis and dying, comparable to the effects of Guanidine Hydrochloride, indicating that PC can protect newborn mice from lethal EV71 challenge. In contrast to Guanidine Hydrochloride, PC can block both EV71 binding and entry processes, suggesting that it may also be used for prophylaxis of viral infection. Besides that, the intramuscular administration of drugs in neonatal mice can better simulate the drug administration in infants and possesses high bioavailability and avoidance of first-pass effect. Thus, PC may be used for prevention and treatment of EV71 infection by intramuscular administration in the future.

In summary, PC possessed anti-EV71 activities both *in vitro* and *in vivo* with low toxicity. PC can block EV71 infection via targeting VP1 protein to interfere with the interaction between VP1 and its receptor SCARB2. Further studies of the inhibition of PC against other EV71 strains in animal models and clinical research will be required to advance it for drug development. Nevertheless, PC merits further studies as a novel antiviral agent for prophylaxis and therapy of EV71 infection.

## Authors’ contributions

Conceptualization, W.W., C.H., Funding acquisition, W.W, C.H., Investigation, J.Q.S., X.Y.M., Data analysis, J.Q.S., L.S.S., Y.Z., Writing, J.Q.S., W.W., Review and editing, C.H., W.W.

## CRediT authorship contribution statement

**Jiqin Sun:** Investigation, Methodology, Formal analysis, Writing – original draft. **Xiaoyao Ma:** Investigation, Validation, Data curation. **Lishan Sun:** Methodology, Formal analysis. **Yang Zhang:** Formal analysis. **Cui Hao:** Conceptualization, Writing – review & editing, Funding acquisition. **Wei Wang:** Conceptualization, Supervision, Writing – review & editing, Funding acquisition.

## Declaration of Competing Interest

The authors declare no conflict of interests.

## Data Availability

Data will be made available on request.

## References

[bib0001] Aswathyraj S., Arunkumar G., Alidjinou E.K., Hober D. (2016). Hand, foot and mouth disease (HFMD): emerging epidemiology and the need for a vaccine strategy. Med. Microbiol. Immunol..

[bib0002] Chen H., Wang W., Yu S., Wang H., Tian Z., Zhu S. (2022). Procyanidins and their therapeutic potential against oral diseases. Molecules.

[bib0003] Dai J., Wang G., Li W., Zhang L., Yang J., Zhao X., Chen X., Xu Y., Li K. (2012). High-throughput screening for anti-influenza A virus drugs and study of the mechanism of procyanidin on influenza A virus-induced autophagy. J. Biomol. Screen..

[bib0004] Dai W., Wu Y., Bi J., Wang S., Li F., Kong W., Barbier J., Cintrat J.C., Gao F., Gillet D., Su W., Jiang C. (2018). Antiviral effects of ABMA against herpes simplex virus type 2 in vitro and in vivo. Viruses.

[bib0005] Dang M., Wang X., Wang Q., Wang Y., Lin J., Sun Y., Li X., Zhang L., Lou Z., Wang J., Rao Z. (2014). Molecular mechanism of SCARB2-mediated attachment and uncoating of EV71. Protein Cell.

[bib0006] Fauvelle C., Lambotin M., Heydmann L., Prakash E., Bhaskaran S., Vishwaraman M., Baumert T.F., Moog C. (2017). A cinnamon-derived procyanidin type A compound inhibits hepatitis C virus cell entry. Hepatol. Int..

[bib0007] Gil-Cardoso K., Comitato R., Gines I., Ardevol A., Pinent M., Virgili F., Terra X., Blay M. (2019). Protective effect of proanthocyanidins in a rat model of mild intestinal inflammation and impaired intestinal permeability induced by LPS. Mol. Nutr. Food Res..

[bib0008] Jin Y., Zhang R., Wu W., Duan G. (2018). Antiviral and inflammatory cellular signaling associated with enterovirus 71 infection. Viruses.

[bib0009] Ku Z., Ye X., Shi J., Wang X., Liu Q., Huang Z. (2015). Single neutralizing monoclonal antibodies targeting the VP1 GH loop of enterovirus 71 inhibit both virus attachment and internalization during viral entry. J. Virol..

[bib0010] Li J., Lin A., Yu C., Zhang Z., Xu D., Hu W., Liu L., Wang S., Nie X., Sun W., Gai Z., Chen Z. (2015). Association of enterovirus 71 encephalitis with the interleukin-8 gene region in Chinese children. Infect. Dis. (Lond.).

[bib0011] Li W., Xu C., Hao C., Zhang Y., Wang Z., Wang S., Wang W. (2020). Inhibition of herpes simplex virus by myricetin through targeting viral gD protein and cellular EGFR/PI3K/Akt pathway. Antiviral Res..

[bib0012] Liao Y.W., Ho B.C., Chen M.H., Yu S.L. (2019). Host relieves lnc-IRAK3-3-sequestered miR-891b to attenuate apoptosis in enterovirus 71 infection. Cell. Microbiol..

[bib0013] Lin W.Y., Yu Y.J., Jinn T.R. (2019). Evaluation of the virucidal effects of rosmarinic acid against enterovirus 71 infection via *in vitro* and *in vivo* study. Virol. J..

[bib0014] Liu Y., Yin W., Wang J., Lei Y., Sun G., Li W., Huang Z., Guo M. (2019). KRAB-zinc finger protein ZNF268a deficiency attenuates the virus-induced pro-inflammatory response by preventing IKK complex assembly. Cells.

[bib0015] Lu Y., Gao Z., Liu C., Long M., Yang L., Li R., Dong K., Zhang H. (2021). Integrative analysis of lncRNA-miRNA-mRNA-associated competing endogenous RNA regulatory network involved in EV71 infection. Am. J. Transl. Res..

[bib0016] Maroli N., Bhasuran B., Natarajan J., Kolandaivel P. (2022). The potential role of procyanidin as a therapeutic agent against SARS-CoV-2: a text mining, molecular docking and molecular dynamics simulation approach. J. Biomol. Struct. Dyn..

[bib0017] Peng H., Shi M., Zhang L., Li Y., Sun J., Zhang L., Wang X., Xu X., Zhang X., Mao Y., Ji Y., Jiang J., Shi W. (2014). Activation of JNK1/2 and p38 MAPK signaling pathways promotes enterovirus 71 infection in immature dendritic cells. BMC Microbiol..

[bib0018] Plevka P., Perera R., Cardosa J., Kuhn R.J., Rossmann M.G. (2012). Crystal structure of human enterovirus 71. Science.

[bib0019] Rasti M., Khanbabaei H., Teimoori A. (2019). An update on enterovirus 71 infection and interferon type I response. Rev. Med. Virol..

[bib0020] Rauf A., Imran M., Abu-Izneid T., Iahtisham Ul H., Patel S., Pan X., Naz S., Sanches Silva A., Saeed F., Rasul Suleria H.A. (2019). Proanthocyanidins: a comprehensive review. Biomed. Pharmacother..

[bib0021] Ruan Y., Jin Q., Zeng J., Ren F., Xie Z., Ji K., Wu L., Wu J., Li L. (2020). Grape seed proanthocyanidin extract ameliorates cardiac remodelling after myocardial infarction through PI3K/AKT pathway in mice. Front. Pharmacol..

[bib0022] Smither S.J., Lear-Rooney C., Biggins J., Pettitt J., Lever M.S., Olinger G.G. (2013). Comparison of the plaque assay and 50% tissue culture infectious dose assay as methods for measuring filovirus infectivity. J. Virol. Method..

[bib0023] Swain S.K., Gadnayak A., Mohanty J.N., Sarangi R., Das J. (2022). Does enterovirus 71 urge for effective vaccine control strategies? Challenges and current opinion. Rev. Med. Virol..

[bib0024] Wang L., Dai Y., Cheng J., Sun C., Chen Y., Cui D. (2021). Analysis of the complete genomes of enterovirus 71 subtypes in China. Can. J. Infect. Dis. Med. Microbiol..

[bib0025] Wang W., Yin R., Zhang M., Yu R., Hao C., Zhang L., Jiang T. (2017). Boronic acid modifications enhance the anti-influenza a virus activities of novel quindoline derivatives. J. Med. Chem..

[bib0026] Yao C., Hu K., Xi C., Li N., Wei Y. (2019). Transcriptomic analysis of cells in response to EV71 infection and 2A(pro) as a trigger for apoptosis via TXNIP gene. Genes Genom..

[bib0027] Zhang Y.X., Huang Y.M., Li Q.J., Li X.Y., Zhou Y.D., Guo F., Zhou J.M., Cen S. (2017). A highly conserved amino acid in VP1 regulates maturation of enterovirus 71. PLoS Pathog..

[bib0028] Zhang Z., Dong Z., Wei Q., Carr M.J., Li J., Ding S., Tong Y., Li D., Shi W. (2017). A neonatal murine model of coxsackievirus A6 infection for evaluation of antiviral and vaccine efficacy. J. Virol..

[bib0029] Zhao S., Zhang L., Yang C., Li Z., Rong S. (2019). Procyanidins and Alzheimer's disease. Mol. Neurobiol..

[bib0030] Zhou D., Zhao Y., Kotecha A., Fry E.E., Kelly J.T., Wang X., Rao Z., Rowlands D.J., Ren J., Stuart D.I. (2019). Unexpected mode of engagement between enterovirus 71 and its receptor SCARB2. Nat. Microbiol..

